# Risk of immune reconstitution inflammatory syndrome with integrase inhibitors versus other classes of antiretrovirals: a systematic review and meta-analysis of randomised trials

**DOI:** 10.1097/QAI.0000000000002937

**Published:** 2022-06-01

**Authors:** Ying Zhao, Ameer Hohlfeld, Phiona Namale, Graeme Meintjes, Gary Maartens, Mark E Engel

**Affiliations:** a Department of Medicine, University of Cape Town, Cape Town, South Africa; b Wellcome Centre for Infectious Diseases Research in Africa, Institute of Infectious Disease and Molecular Medicine, University of Cape Town, Cape Town, South Africa; c Cochrane South Africa, South African Medical Research Council, Cape Town, South Africa; d Division of Clinical Pharmacology, Department of Medicine, University of Cape Town, Cape Town, South Africa

**Keywords:** Antiretroviral therapy, HIV, integrase inhibitors, immune reconstitution inflammatory syndrome

## Abstract

**Background:**

Integrase strand transfer inhibitors (InSTIs) decrease HIV plasma viral load faster than other antiretroviral classes. More rapid viral load decline has been associated with higher risk of immune reconstitution inflammatory syndrome (IRIS). There are conflicting reports on the association between InSTI and IRIS. We performed a systematic review and meta-analysis to compare the risk of IRIS among treatment-naïve HIV-positive patients starting InSTI versus non-InSTI regimens.

**Methods:**

We searched PubMed, Scopus, Web of Science, Africa-Wide, and Cochrane databases from earliest available date to 26 November 2021, for randomised controlled trials (RCTs) having intervention arms with InSTI versus control arms without InSTI in patients initiating first-line antiretroviral therapy. The primary outcome was relative risk (RR) of IRIS, while the secondary outcome was RR of paradoxical tuberculosis-associated IRIS (TB-IRIS). Data were combined by random-effects meta-analysis according to the Mantel-Haenszel method. The protocol for this study is registered with PROSPERO, CRD42020213976.

**Results:**

We included 14 RCTs comprising 8696 participants from six continents for the primary outcome of IRIS, and a subset of 674 participants (from three RCTs) for the secondary outcome of paradoxical TB-IRIS. Risk of IRIS was similar between InSTI and non-InSTI regimens (RR, 0.93, 95% confidence interval [CI], 0.75 – 1.14). There was a trend towards a lower risk of paradoxical TB-IRIS with InSTI versus efavirenz regimens that was not statistically significant (RR, 0.64, 95% CI, 0.34 – 1.19).

**Conclusions:**

In this meta-analysis among treatment-naïve patients commencing first-line antiretroviral therapy, InSTI regimens were not associated with higher risk of IRIS.

## Introduction

Dolutegravir, an integrase strand transfer inhibitor (InSTI), is replacing efavirenz as the preferred drug in first-line antiretroviral therapy (ART) in low- to middle-income countries (LMICs) due to its high genetic barrier to resistance, improved tolerability, and reduced cost as a generic fixed-dose combination with tenofovir and lamivudine ^1-3^.

InSTIs decrease plasma HIV viral load faster than other antiretroviral classes ^4-6^. All InSTIs are associated with similarly rapid HIV viral load decline ^7^. Rapid reduction in HIV viraemia does not appear to confer clinical benefit ^5,8^. However, rapid recovery of immune function during first-line ART may increase the risk of immune reconstitution inflammatory syndrome (IRIS), an immunopathological reaction characterised by paradoxical worsening of treated opportunistic infections or unmasking of subclinical infections. An association between more rapid HIV viral load decline and IRIS has been observed in certain cohort studies ^9-11^.

Tuberculosis is the commonest cause of morbidity and mortality in people living with HIV (PLWH) ^12,13^. Paradoxical tuberculosis-associated IRIS (TB-IRIS) can manifest shortly after ART initiation, occurring in 18% of patients receiving antituberculosis treatment according to a pooled estimate, with incidences ranging from 4 – 54% in individual studies ^14^. In sub-Saharan Africa, ART is often started when PLWH present for care with advanced immunosuppression (CD4 count below 100 cells/mm^3^) ^15,16^, which is an important risk factor for TB-IRIS ^14,17^. In populations with high prevalence of TB-IRIS risk factors (low CD4 counts and short interval between antituberculosis treatment and ART initiation), the incidence of TB-IRIS may exceed 50% ^18,19^. Paradoxical TB-IRIS is associated with significant morbidity, with 25% requiring hospitalisation ^14^.

InSTIs were associated with a two- to three-fold increased risk of IRIS in cohort studies, but only a few TB-IRIS events were reported ^20-22^. The most recent systematic review published on IRIS risk in randomised controlled trials (RCTs) ^23^ reported only 13 cases of IRIS; furthermore, the included RCTs excluded patients with Centers for Disease Control and Prevention (CDC) stage C disease at baseline, who have a higher risk of IRIS and IRIS-associated death ^24^. Since then, there have been several RCTs published on InSTI regimens that have enrolled patients with CDC stage C disease and HIV-associated tuberculosis. Therefore, we conducted a systematic review and meta-analysis of RCTs, comprising ART-naïve participants randomised to InSTI versus other antiretroviral classes, to provide robust evidence on the association of InSTI with IRIS and paradoxical TB-IRIS.

## Study Population and Methods

### Search strategy and selection criteria

The protocol for this study followed PRISMA guidelines and was approved by the Human Research Ethics Committee at the University of Cape Town (Ref 682/2020). We registered the study protocol on PROSPERO (CRD42020213976).

The main search comprises individual searches using detailed medical subject heading (MeSH) terms for “HIV”, “AIDS”, “integrase inhibitor”, “dolutegravir”, “raltegravir”, “elvitegravir”, “bictegravir”, “cabotegravir”, and “RCT”, and related terms for relevant studies published in Medline (accessed via PubMed), Scopus, Web of Science, Africa-Wide, and Cochrane databases from earliest available date to 26 November 2021, without language restriction. The full search strategy is shown in Table 1, Supplemental Digital Content. We searched for grey literature, including the 2014-2021 proceedings from the Conference on Retroviruses and Opportunistic Infections (CROI) and the International AIDS Society Annual Conference. In addition, we searched the database www.clinicaltrials.gov and reviewed bibliographies of all included studies for potentially eligible studies. Search results were managed using the Rayyan platform ^25^. Two reviewers (YZ and PN) independently screened titles and abstracts in duplicate. YZ and PN evaluated full texts of potentially relevant articles independently and in duplicate using a standardised form. YZ and PN compiled and compared their own list of eligible studies. Any disagreement on eligibility was resolved through discussion and consultation with a third reviewer (GaM).

### Eligibility criteria

Eligible studies were RCTs that enrolled adults (age ≥18 years) who were HIV-positive and initiating first-line ART, with intervention arms containing InSTI versus control arms with other antiretroviral classes. We included studies in which InSTI was used in combination with any other antiretrovirals (nucleoside reverse transcriptase inhibitor, non-nucleoside reverse transcriptase inhibitor, or protease inhibitor) and if the control arm did not have InSTI. RCTs were included if IRIS events were reported. We excluded observational studies due to potential bias in InSTI use, studies with a single-arm or cross-over design, studies with InSTI in all treatment arms, and studies evaluating the switch to InSTI regimens in virologically suppressed patients.

### Data extraction and quality assessment

Two review authors (YZ and PN) collected data from text, tables, and figures of each eligible study onto a pre-designed standardised data extraction form (File 1, Supplemental Digital Content) independently, and in duplicate, and cross-checked results. Any disagreement in data extraction was resolved by discussion between the review authors. We extracted numbers of IRIS events reported by primary studies among participants initiating ART, who were at risk for IRIS. IRIS events reported as adverse events were also collected from the database www.clinicaltrials.gov and supplementary appendices of trial publications whenever available. Study authors were contacted in the case of missing data.

Studies were critically appraised to evaluate the risk of bias using the revised Cochrane risk of bias tool for randomised trials (RoB 2) ^26^. The effect of interest was the effect of assignment to the intervention at baseline (the “Intention-to-treat effect”). We assessed the risk of bias for outcomes reported in the included studies that we specified as outcomes for the current review (IRIS and paradoxical TB-IRIS). Bias was assessed in five distinct domains: bias arising from the randomisation process; bias due to deviations from intended interventions; bias due to missing outcome data; bias in measurement of the outcome; and bias in selection of the reported result. We used the proposed RoB 2 algorithms to report judgements for each domain and overall as low risk of bias, some concerns, or high risk of bias. Two review authors (YZ and AH) assessed the risk of bias for each outcome independently and in duplicate. Any disagreement in judgement was resolved by discussion between the review authors.

We used the Grading of Recommendations Assessment, Development and Evaluation (GRADE) approach to present the overall quality of evidence for the following outcomes: IRIS and paradoxical TB-IRIS ^27^. Two review authors (YZ and AH) independently rated the certainty of evidence for each outcome as high, moderate, low, or very low. We considered five criteria related to internal validity (risk of bias, inconsistency, imprecision, publication bias) and external validity (indirectness). Any disagreement in judgement was resolved by discussion between the review authors. We used the GRADEpro GDT software to create a “Summary of findings” table. ^28^

### Statistical analysis

We report proportions with IRIS events as point estimates with 95% confidence intervals (CIs). As none of the studies were specifically designed to assess IRIS as a primary outcome, our denominator for calculating proportions with IRIS events was defined as the total number of participants enrolled who initiated ART and were at risk of developing IRIS. Relative risks (RRs) for the comparison between InSTI and other antiretroviral classes were calculated for IRIS events and reported with corresponding 95% CIs. We conducted an overall meta-analysis of IRIS events across studies, as well as sub-group meta-analyses of IRIS events by individual InSTI drugs (dolutegravir, raltegravir, and elvitegravir). Meta-analyses were performed according to the Mantel-Haenszel method applying the random-effects models. Studies that enrolled participants with HIV-associated tuberculosis were combined in a sub-group meta-analysis to assess the secondary outcome of paradoxical TB-IRIS. We evaluated heterogeneity using the Cochrane’s Chi^2^ test (significant if *P* < 0.10) and the *I*^2^ statistic test (>50% indicative of substantial heterogeneity) ^29^.

## Results

### Study characteristics

**Figure 1:**
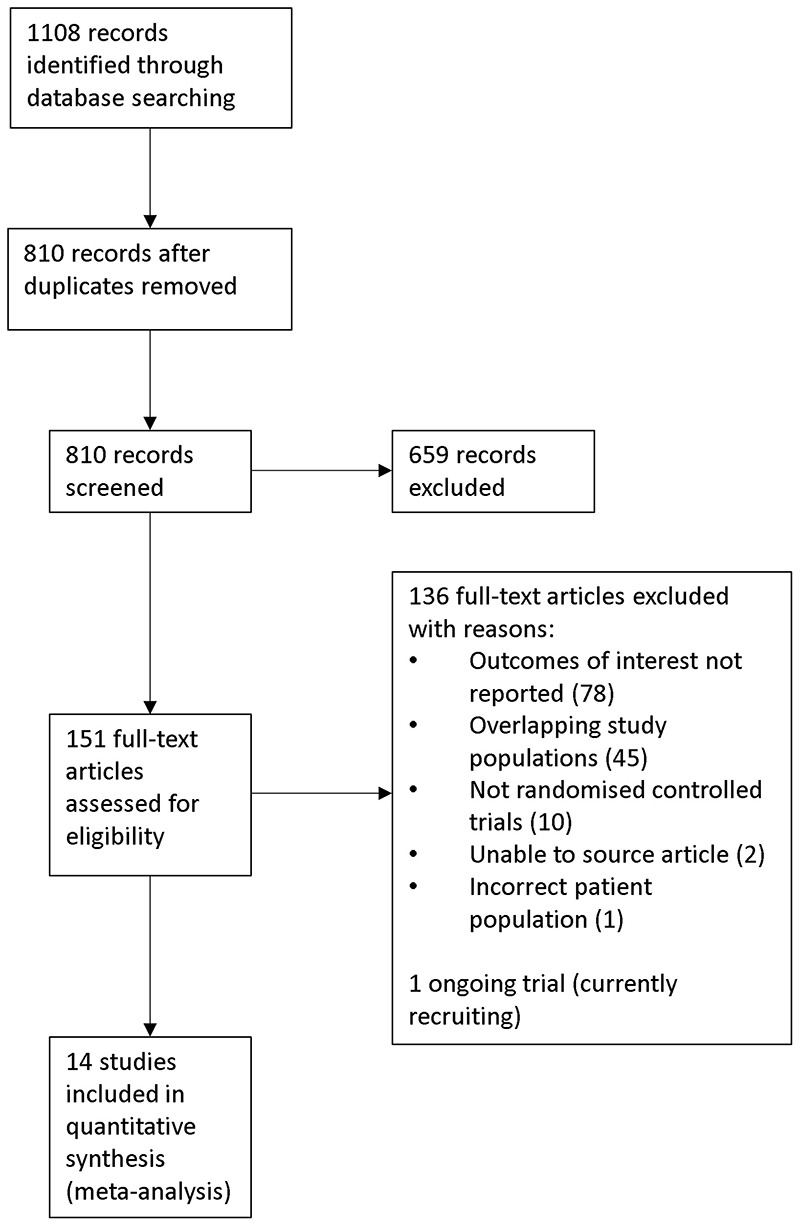
PRISMA flow diagram

We identified 1108 records from five databases, 151 of which were eligible for full-text review. Fourteen RCTs were included in our meta-analysis (Figure 1).

The characteristics of the included studies are summarised in Table 2, Supplemental Digital Content. Most studies (n = 11) were multinational, and participants were recruited from Africa (n = 7), Europe (n = 6), North America (n = 5), South America (n = 5), Asia (n = 5), and Australia (n = 2). Two studies ^5,30^ enrolled children (aged ≥5 years) and/or adolescents (aged ≥13 years) in addition to adult participants. Both studies were included in our analysis as few participants were aged <18 years: 72/1805 (4.0%) participants were aged 5 – 17 years in REALITY ^5^ and 14/1053 (1.3%) aged 13 – 18 years in ADVANCE ^30^. Dolutegravir was the InSTI drug used in the intervention arms in seven studies ^4,8,30-34^, raltegravir in six studies ^5,35-39^, and elvitegravir in one study ^40^.

The primary outcome of included studies was proportion with virologic suppression ^4,8,30,32-38,40^, time to virologic or clinical failure ^39^, all-cause mortality ^5^, or median CD4 count increase at week 48 ^31^. Seven trials defined IRIS as a secondary outcome ^5,8,31,32,35,36,39^, including four that specifically defined TB-IRIS as a secondary outcome ^5,32,35,36^. The case definition proposed by French ^41^ was used in the diagnosis of IRIS in two studies ^5,8^ and the International Network for the Study of HIV-associated IRIS (INSHI) case definition ^42^ in the diagnosis of paradoxical TB-IRIS in five studies ^5,8,32,35,36^. Nine studies did not specify the case definition used for IRIS adjudication ^4,30,31,33,34,37-40^. An independent endpoint review committee adjudicated IRIS events in six studies ^5,8,30,32,35,36^, three of which specified that adjudication was blinded to treatment arms ^5,8,32^.

Full safety data were available for only two studies as supplementary appendices to the main publications of the trials ^4,33^. The safety data reported by the other studies included serious adverse events (SAEs), adverse events leading to drug discontinuation, Grade 3 and 4 adverse events, and adverse events that occurred with a frequency threshold of 5%. Therefore, IRIS events classified as Grade 1 and 2 adverse events or occurred in <5% of participants and not reported by primary studies could not be assessed in this meta-analysis. The Late Presenter Treatment Optimisation (LAPTOP) study is ongoing and could not be included in this analysis (estimated date of completion in December 2021) ^43^.

### Risk of bias in included studies

The RoB 2 judgements for all domains, and overall for the primary and secondary outcomes, are summarised in Table 3, Supplemental Digital Content. Six studies were evaluated as having some concerns for the overall risk of bias for the primary outcome of IRIS ^8,30,35,36,38,40^. Six studies were at overall high risk of bias for IRIS ^4,31,33,34,37,39^. The main reason for a study being assessed as having a high risk of bias was measurement of the outcome. Three studies contributing to the secondary outcome of paradoxical TB-IRIS were at overall low risk of bias ^32^ or had some concerns ^35,36^.

### Primary outcome: Risk of IRIS

**Figure 2:**
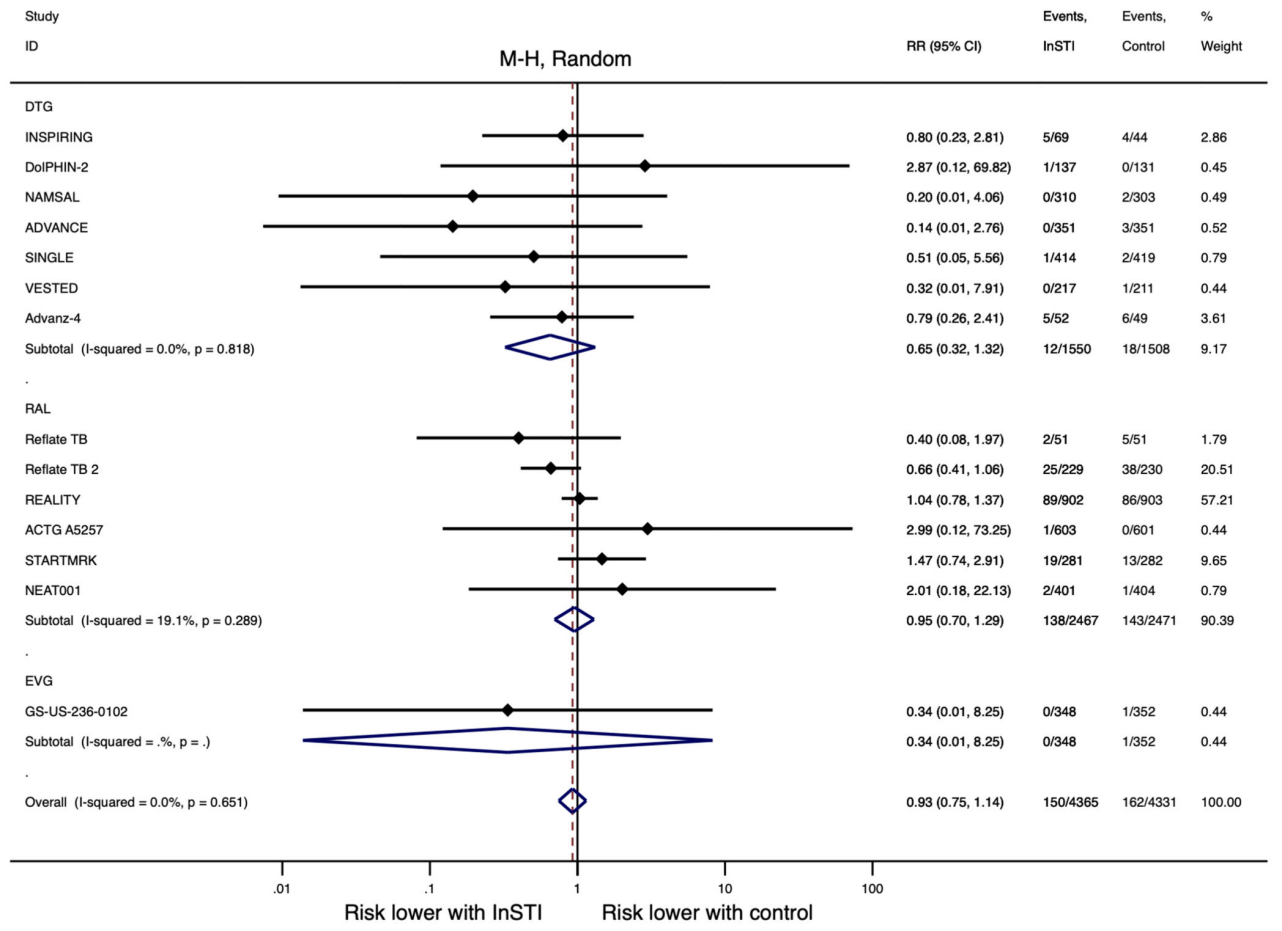
Meta-analysis of IRIS in randomized controlled trials of InSTI IRIS, immune reconstitution inflammatory syndrome; InSTI, integrase strand transfer inhibitor; DTG, dolutegravir; RAL, raltegravir; EVG, elvitegravir; M-H, Mantel-Haenszel; RR, risk ratio; CI, confidence interval.

**Figure 3:**
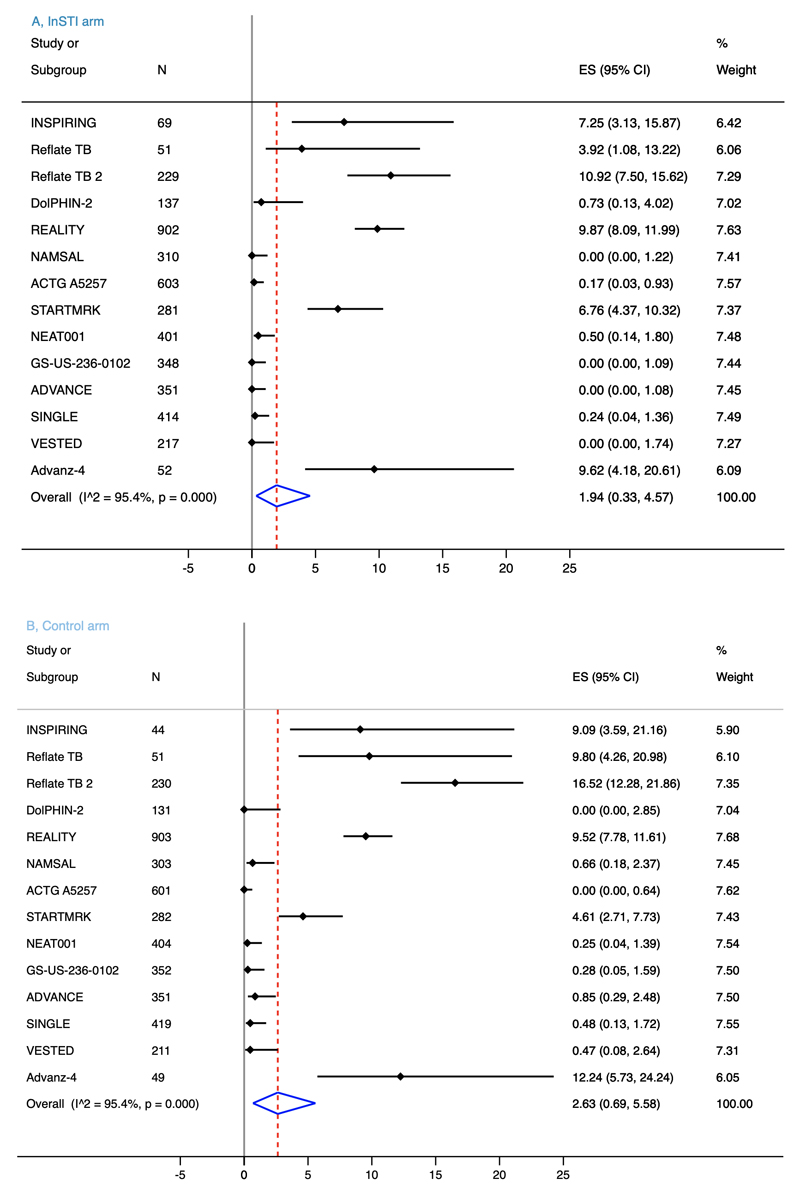
Forest plots of proportion of patients with IRIS on InSTI regimens (a) and non-InSTI regimens (b) IRIS, immune reconstitution inflammatory syndrome; InSTI, integrase strand transfer inhibitor; ES, effect size; CI, confidence interval.

There is probably little to no difference in IRIS risk between InSTI and non-InSTI regimens (RR, 0.93, 95% CI, 0.75 – 1.14, *I*^2^ = 0.0%, 8696 participants, moderate-certainty evidence, Figure 2). Risk of IRIS was similar between individual member of InSTI class and non-InSTI regimens: dolutegravir (RR, 0.65, 95% CI, 0.32 – 1.32, *I*^2^ = 0.0%, 7 studies, 3058 participants), raltegravir (RR, 0.95, 95% CI, 0.70 – 1.29, *I*^2^ = 19.1%, 6 studies, 4938 participants), and elvitegravir (RR, 0.34, 95% CI, 0.01 – 8.25, 1 study, 700 participants). Heterogeneity among studies was low for the primary outcome (*I*^2^ = 0.0%, *P*_heterogeneity_ = 0.651). We downgraded the certainty of evidence one level for serious risk of bias. The evidence profile is presented in the “Summary of findings” table (Table 4, Supplemental Digital Content). IRIS events were reported for 1.94% (*n* = 4365, 95% CI, 0.33 – 4.57) participants on InSTI regimens versus 2.63% (*n* = 4331, 95% CI, 0.69 – 5.58) on non-InSTI regimens (Figure 3).

The Reflate TB 2 study, a multinational trial recruiting participants with HIV-associated tuberculosis, reported the highest incidence of IRIS: 11% (25/229) participants on raltegravir regimen versus 17% (38/230) on efavirenz regimen developed IRIS events ^36^. IRIS event rates were low (<1%) in seven studies ^4,8,33,34,37,39,40^. Five studies reported an incidence of IRIS between 1 and 10% ^5,30,32,35,38^. Proportions with IRIS reported in included studies are shown in Table 5, Supplemental Digital Content. We did not observe substantial changes to our overall estimate of IRIS risk between InSTI and non-InSTI regimens with a sub-group meta-analysis excluding studies that reported <5% IRIS events (RR, 0.93, 95% CI, 0.72 – 1.19, *I*^2^ = 9.1%, 6 studies, 3143 participants).

Eight IRIS events were reported as SAEs (2 on raltegravir regimen and 1 on efavirenz regimen in STARTMRK ^44^, 1 on dolutegravir regimen and 1 on efavirenz regimen in INSPIRING ^32^, 1 on raltegravir regimen in ACTG A5257 ^37^, 1 on dolutegravir regimen in DolPHIN-2 ^8^, and 1 on efavirenz regimen in GS-US-236-0102 ^40^). One IRIS event led to discontinuation of ART regimen (raltegravir regimen of STARTMRK ^44^). In the REALITY study, 36/902 (4.0%) participants on raltegravir-intensified regimen died as result of IRIS, compared with 31/903 (3.4%) on standard ART regimen that did not contain raltegravir ^5^. Fatal IRIS occurred in 2/303 participants on efavirenz regimen in NAMSAL, manifesting as pulmonary tuberculosis and Kaposi sarcoma ^33^. In Reflate TB 2, TB-IRIS was the cause of death in two participants: 1/229 on raltegravir regimen and 1/230 on efavirenz regimen ^36^.

One study reported median time from ART initiation to IRIS occurrence as 3.4 (interquartile range, 2.0 – 6.3) weeks, with rates declining from the third week on ART ^5^. Four late-occurring IRIS events were reported in STARTMRK, with two cases on raltegravir regimen and two cases on efavirenz regimen developing after 48 weeks ^38^.

### Secondary outcome: Risk of paradoxical TB-IRIS

Three studies (INSPIRING, Reflate TB, and Reflate TB 2) enrolled 674 participants at risk for paradoxical TB-IRIS (starting ART after a diagnosis of HIV-associated tuberculosis while on antituberculosis treatment) ^32,35,36^. The INSPIRING study compared a twice daily dolutegravir regimen to efavirenz regimen ^32^. Both Reflate TB studies compared raltegravir and efavirenz; Reflate TB included two raltegravir regimens using two different dosing strategies ^35^.

**Figure 4:**
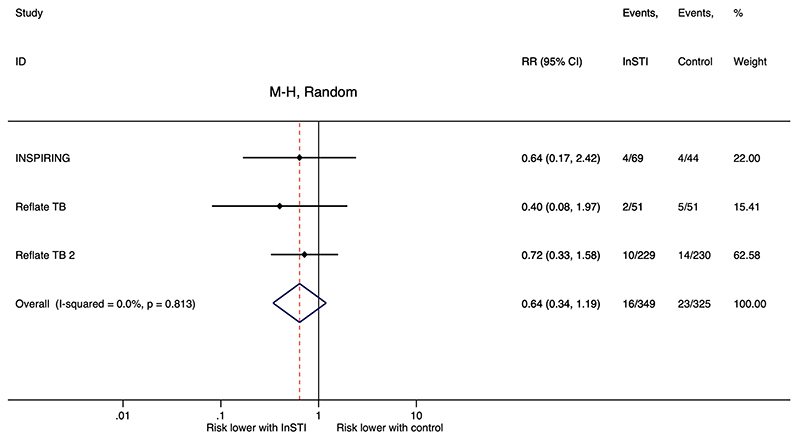
Meta-analysis of paradoxical TB-IRIS in randomized controlled trials of InSTI TB-IRIS, tuberculosis-associated immune reconstitution inflammatory syndrome; InSTI, integrase strand transfer inhibitor; M-H, Mantel-Haenszel; RR, risk ratio, CI, confidence interval.

There may be a reduced risk of paradoxical TB-IRIS among participants initiating InSTI regimens than those initiating efavirenz regimens, but this was not statistically significant (RR, 0.64, 95% CI, 0.34 – 1.19, *I*^2^ = 0.0%, 674 participants, low-certainty evidence, Figure 4). Heterogeneity between the studies was low for this secondary outcome (*I*^2^ = 0.0%, *P*_heterogeneity_ = 0.813). We downgraded the certainty of evidence due to serious risk of bias and imprecision. The evidence profile is presented in the “Summary of findings” table (Table 4, Supplemental Digital Content). One SAE was attributed to paradoxical TB-IRIS (dolutegravir regimen in INSPIRING) ^32^. Two deaths due to paradoxical TB-IRIS were reported: 1 on efavirenz regimen and 1 on raltegravir regimen in Reflate TB 2 ^36^.

## Discussion

In our meta-analysis of data from RCTs across six continents, we found no association between InSTI regimens and risk of IRIS. We found a trend towards a lower risk of paradoxical TB-IRIS with InSTI regimens compared with efavirenz regimens among patients with HIV-associated tuberculosis initiating ART, but precision was lacking for this secondary outcome. It is reassuring that initiating InSTI regimens did not increase the risk of IRIS in our systematic review that included trial participants across eight sub-Saharan African countries, therefore supporting the current move to first-line InSTI regimens in LMICs.

Our finding that InSTI regimens were not associated with risk of IRIS is contrary to the results from observational cohort studies. In the Dutch ATHENA cohort, 32% of patients starting InSTI and 18% starting non-InSTI regimens developed IRIS (odds ratio [OR] 2.17, 95% CI, 1.45 – 3.25) ^20^. Severe IRIS leading to hospitalisation occurred in 3% of patients on InSTI and 1.5% on non-InSTI regimens in a multicentre French cohort (RR 1.99, 95% CI, 1.09 – 3.47) ^21^. Psichogiou et al. reported InSTI use an independent risk factor for IRIS (OR 2.89, 95% CI, 1.26 – 6.64) ^22^. We found a trend towards a lower risk of paradoxical TB-IRIS with InSTI regimens compared with efavirenz regimens among patients with HIV-associated tuberculosis initiating ART. However, an increased risk of paradoxical TB-IRIS was reported in patients with newly diagnosed tuberculosis initiating InSTI regimen in a retrospective cohort study (OR 3.33, 95% CI, 1.01 – 11.1) ^45^. Bias and unmeasured confounders may have contributed to the observed association with IRIS in observational cohort studies. First, diagnoses of IRIS rely on the detection of clinical features that fulfil case definitions and are challenging due to the lack of specific diagnostic tests. An increasing awareness of a possible association between InSTI and IRIS and the nature of an unblinded study and retrospective chart review may lead to ascertainment bias. Second, patients presenting with specific opportunistic infections were more likely to receive InSTI regimens to avoid drug-drug interactions with concomitant medications in one study ^20^, resulting in patients most at risk for IRIS being preferentially channelled to InSTI regimens.

InSTI regimens result in more rapid HIV viral load declines ^4-6^. The hypothesis that IRIS would be more common with InSTI regimens was based on cohort studies reporting the rapid decline in HIV viral load after ART initiation an independent risk factor for paradoxical TB-IRIS ^10,11^. In a retrospective observational study, patients who developed IRIS had more marked decline in viral load within 90 days of starting ART (*P* < 0.001), and a viral load decrease of 2 log10 copies/mL at 90 days on ART was associated with a 3.7-fold increase in risk of developing IRIS (95% CI, 1.55 – 8.64; *P* = 0.003) ^9^. Similarly, in a study of immunologic predictors of TB-IRIS, a viral load decline ≥4 log10 copies/mL at week 12 was associated with TB-IRIS (OR 2.56, 95% CI, 1.00 – 6.59) ^46^. However, our review of RCTs found no excess of IRIS events observed on InSTI regimens, despite the substantially faster viral load decline with InSTI regimens as demonstrated with raltegravir-intensified regimen in the REALITY study ^5^. Our findings suggest that rapid viral load decline with ART initiation might not be an independent risk factor for IRIS. Further research into the immunological mechanisms underlying IRIS is needed to better predict the development of this condition.

Our study serves to update an earlier systematic review which excluded patients with CDC stage C disease and HIV-associated tuberculosis ^23^. Nevertheless, this review has important limitations. First, there was likely under-reporting of IRIS events as none of the RCTs assessed IRIS as a primary outcome and due to restrictions used by RCTs in the reporting of safety results. One study reported only fatal IRIS events ^33^ and three studies reported IRIS events that met criteria for an SAE ^8,37,40^. Two studies reported only Grade 3 and 4 paradoxical TB-IRIS events ^35,36^. Our pooled estimates of IRIS events were low compared with other meta-analyses ^14,47^ and likely reflected an under-ascertainment of IRIS events. However, under ascertainment was expected to be similar in participants randomised to InSTI and non-InSTI regimens. Our findings may thus reflect the more clinically significant and severe spectrum of IRIS events. Second, adjudication of IRIS event was not masked to treatment allocation in some studies and case definitions used to ascertain IRIS events differed across studies. Third, we did not have individual participant data to explore risk of IRIS in sub-groups such as patients with low CD4 counts.

A further limitation was our lack of precision for the risk of paradoxical TB-IRIS. Heterogeneity between the studies was low for this secondary outcome, but the associated 95% CI was wide due to imprecision in meta-analysis of few studies. It is also plausible that TB-IRIS was uncommon because patients at highest risk for TB-IRIS were underrepresented in the RCTs included in our sub-group analysis. Low CD4 counts and short intervals between antituberculosis treatment and ART initiation are risk factors most consistently associated with TB-IRIS ^14,48^. Patients with advanced immunosuppression (CD4 count <50 cells/mm^3^) were excluded from INSPIRING and underrepresented in Reflate TB (20%) and Reflate TB 2 (33%) ^32,35,36^. Median time to starting ART was over four weeks in two studies (5.9 weeks in Reflate TB ^35^; 35 and 33.5 days on dolutegravir and efavirenz regimens, respectively, in INSPIRING ^32^).

## Conclusions

We found no increased risk of IRIS with InSTI regimens in this meta-analysis of RCTs. We do, however, highlight the need for further studies, including RCTs with less restrictive eligibility criteria, to determine whether InSTI increase the risk and/or severity of paradoxical TB-IRIS in high-risk patients (CD4 counts <50 cells/mm^3^ and starting ART within four weeks of antituberculosis treatment). Notwithstanding the limitations of the study, findings from this meta-analysis provide additional evidence to support the routine use of InSTI in first-line ART regimens in LMICs.

## Supplementary Material

Supplementary material
