# Supplementary material for: Risk of immune reconstitution inflammatory syndrome with integrase inhibitors versus other classes of antiretrovirals: a systematic review and meta-analysis of randomised trials
Source: J Acquir Immune Defic Syndr. Author manuscript; Available in PMC 2022 Jun 19. (PMC7612870; doi:10.1097/QAI.0000000000002937)
Supplement: Supplementary material [file EMS141035-supplement-Supplementary_material.docx]

**Table 1. Search strategy**

| **Search** | **MeSH term (modified as needed for use in other databases)** |
| --- | --- |
| #1 | “Human immunodeficiency virus” |
| #2 | “HIV infection” |
| #3 | “HIV seropositivity” |
| #4 | HIV-1 |
| #5 | #1 OR #2 OR #3 OR #4 |
| #6 | “Acquired immunodeficiency syndrome” |
| #7 | “AIDS serodiagnosis” |
| #8 | #6 OR #7 |
| #9 | #5 OR #8 |
| #10 | “Integrase inhibitor” |
| #11 | “HIV integrase inhibitor” |
| #12 | “Integrase strand transfer inhibitor” |
| #13 | Dolutegravir |
| #14 | Raltegravir |
| #15 | Bictegravir |
| #16 | Elvitegravir |
| #17 | Cabotegravir |
| #18 | #10 OR #11 OR #12 OR #13 OR #14 OR #15 OR #16 OR #17 |
| #19 | “Protease inhibitor” |
| #20 | “HIV protease inhibitor” |
| #21 | Darunavir |
| #22 | Atazanavir |
| #23 | Lopinavir |
| #24 | #19 OR #20 OR #21 OR #22 OR #23 |
| #25 | “Reverse transcriptase inhibitors” |
| #26 | “Non-nucleoside reverse transcriptase inhibitor” |
| #27 | Efavirenz |
| #28 | Nevirapine |
| #29 | Doravirine |
| #30 | Rilpivirine |
| #31 | Etravirine |
| #32 | #25 OR #26 OR #27 OR #28 OR #29 OR #30 OR #31 |
| #33 | #24 OR #32 |
| #34 | RCT |
| #35 | “Randomised control* trial” |
| #36 | #34 OR #35 |
| #37 | #9 AND #18 AND #33 AND #36 |
| *MeSH, medical subject heading; HIV, human immunodeficiency virus; AIDS, acquired immunodeficiency syndrome; RCT, randomised controlled trial.* | |

**File 1. Data extraction form**

| **Study Author** |  |
| --- | --- |
| **Title** |  |

1. **General information on the study**

| **Date form completed** |  |
| --- | --- |
| **Name of person extracting data** |  |
| **Corresponding author & contacts** |  |

1. **Study eligibility**

| **Characteristic** | **Review Inclusion Criteria** | **Yes** | **No** | **Unclear** | **Location in text** |
| --- | --- | --- | --- | --- | --- |
| **Participants** | HIV-positive, ART-naïve adults starting first-line regimen |  |  |  |  |
| **Study Design** | Randomised controlled trial |  |  |  |  |
| **Intervention** | Integrase inhibitor-based ART |  |  |  |  |
| **Control** | Protease inhibitor-based ART or  NNRTI-based ART |  |  |  |  |
| **Outcome** | IRIS event reported |  |  |  |  |

| **Final decision on study eligibility** | **Include** | **Exclude** |
| --- | --- | --- |
| **Reason for exclusion** |  | |
| **Notes:** | | |

**DO NOT PROCEED IF STUDY EXCLUDED FROM REVIEW.**

1. **Study population and clinical setting**

|  | **Description** | **Location in text** |
| --- | --- | --- |
| **Country** |  |  |
| **Inclusion criteria (NB: CD4 criteria)** |  |  |
| **Exclusion criteria** |  |  |
| **Number of participants enrolled** |  |  |
| **Adjudication of IRIS (endpoint review committee)** |  |  |
| **Adjudication blinded or not** |  |  |
| **Protocol-defined criteria for IRIS** |  |  |
| **Definition of TB-IRIS used** |  |  |
| **Notes:** | | |

1. **Methods**

|  | **Description** | **Location in text** |
| --- | --- | --- |
| **Primary outcome/endpoint** |  |  |
| **Study design** |  |  |
| **Intervention arm** |  |  |
| **Control arm** |  |  |
| **Randomisation process** |  |  |
| **Notes:** | | |

1. **Assessment of design methodology**

|  | **Description** | **Location in text** |
| --- | --- | --- |
| **Sequence generation** |  |  |
| **Concealment of allocation** |  |  |
| **Blinding** |  |  |
| **Incomplete outcome data** |  |  |
| **Selective outcome reporting** |  |  |
| **Sample size calculation & adequacy** |  |  |
| **Final decision on performance bias** |  |  |
| **Notes (any other source of bias):** | | |

1. **Participant baseline characteristics**

|  | **Intervention arm**  **(n = )** | **Control arm**  **(n = )** | **P-value** |
| --- | --- | --- | --- |
| **Female, n (%)** |  |  |  |
| **Male, n (%)** |  |  |  |
| **Age, median (IQR), years** |  |  |  |
| **CD4 cell count, median (IQR), cells/mm^3^** |  |  |  |
| **CD4 cell counts, <100 cells/mm^3^, n (%)** |  |  |  |
| **CD4 cell counts, >/=100 cells/mm^3^, n (%)** |  |  |  |
| **HIV viral load, median (IQR), log_10_ copies/mL** |  |  |  |
| **HIV viral load, <100 000 copies/mL, n (%)** |  |  |  |
| **HIV viral load, >/=100 000 copies/mL, n (%)** |  |  |  |
| **Received cotrimoxazole prophylaxis, n (%)** |  |  |  |
| **Received TB prophylaxis, n (%)** |  |  |  |

1. **Incidence of IRIS**

|  | **Intervention arm**  **(n = )** | **Control arm**  **(n = )** | **P-value** |
| --- | --- | --- | --- |
| **IRIS events, n (%), [N]** |  |  |  |
| **Grade 3-4 AE attributed to IRIS, n (%), [N]** |  |  |  |
| **Serious AE attributed to IRIS, n (%), [N]** |  |  |  |
| **Drug discontinued due to IRIS, n (%), [N]** |  |  |  |
| **Death attributed to IRIS, n (%), [N]** |  |  |  |
| **IRIS requiring intervention, n (%), [N]** |  |  |  |
| **IRIS requiring corticosteroids, n (%), [N]** |  |  |  |
| **Unmasking IRIS, n (%), [N]** |  |  |  |
| **Paradoxical IRIS, n (%), [N]** |  |  |  |
| **Unmasking TB-IRIS, n (%), [N]** |  |  |  |
| **Paradoxical TB-IRIS, n (%), [N]** |  |  |  |
| **Non-TB-IRIS of known aetiology, n (%), [N]** |  |  |  |
| **Description of aetiology** |  |  |  |
| **IRIS of unknown aetiology, n (%), [N]** |  |  |  |
| **Time from ART start to IRIS occurrence, median (IQR), weeks** |  |  |  |

**Note: Table shows number of participants with one or more episode of IRIS (percentage of participants), [number of IRIS episodes].**

1. **Paradoxical TB-IRIS**

|  | **Intervention arm**  **(n = )** | **Control arm**  **(n = )** | **P-value** |
| --- | --- | --- | --- |
| **Current TB disease, n (%)** |  |  |  |
| **Pulmonary TB, n (%)** |  |  |  |
| **Extrapulmonary, n (%)** |  |  |  |
| **If extrapulmonary, location (n)** |  |  |  |
| **Time from start of TB therapy to ART, median (IQR), weeks** |  |  |  |
| **Grade 3-4 AE attributed to TB-IRIS, n (%)** |  |  |  |
| **Serious AE attributed to TB-IRIS, n (%)** |  |  |  |
| **Drug discontinued due to TB-IRIS, n (%)** |  |  |  |
| **Death attributed to TB-IRIS, n (%)** |  |  |  |
| **TB-IRIS requiring corticosteroids, n (%)** |  |  |  |
| **Time from ART start to TB-IRIS occurrence, median (IQR), weeks** |  |  |  |

**Table 2. Main characteristics of included studies**

| **Study** | **Study duration, weeks** | **Treatment Arms** | **N** | **Age, years, median (IQR)** | **Female, n (%)** | **Baseline CD4 count, cells/mm^3^, median (IQR)** | **Baseline viral load, log_10_ copies/mL, median (IQR)** |
| --- | --- | --- | --- | --- | --- | --- | --- |
| SINGLE | 144 | DTG + ABC/3TC | 414 | 36 (18, 68)^a^ | 67 (16) | 335^c^ | 4.67^c^ |
|  |  | EFV + TDF/FTC | 419 | 35 (18, 85)^a^ | 63 (15) | 339^c^ | 4.70^c^ |
| DolPHIN-2 | 72 weeks postpartum | DTG + TDF/3TC | 125 | 28.0 (5.3)^b^ | 125 (100) | 464 (329, 664) | 4.4 (3.6, 4.7) |
|  |  | EFV + TDF/3TC | 125 | 27.4 (5.1)^b^ | 125 (100) | 414 (265, 581) | 4.6 (3.9, 4.8) |
| Advanz-4 | 48 | DTG + ABC/3TC | 52 | 40 (30, 48) | 8 (13) | 41 (18, 67) | 5.47 (4.79, 6.10) |
|  |  | DRV/r + ABC/3TC | 49 | 41 (34, 46) | 3 (11) | 30 (11, 54) | 5.67 (5.14, 6.12) |
| INSPIRING | 48 | DTG + 2NRTI | 69 | 33 (18, 62) | 30 (43) | 208 (128, 410) | 5.10 (4.74, 5.47) |
|  |  | EFV + 2NRTI | 44 | 32 (20, 50) | 16 (36) | 202 (92, 354) | 5.24 (4.50, 5.67) |
| NAMSAL | 96 | DTG + 3TC/TDF | 310 | 38 (31, 46) | 197 (64) | 289 (157, 452) | 5.3 (4.8, 5.8) |
|  |  | EFV + 3TC/TDF | 303 | 36 (29, 43) | 207 (68) | 271 (147, 427) | 5.3 (4.7, 5.8) |
| ADVANCE | 96 | DTG + TAF/FTC | 351 | 33 (7.8)^b^ | 214 (61) | 349 (225.3)^a^ |  |
|  |  | EFV + TDF/FTC | 351 | 32 (7.4)^b^ | 201 (57) | 337 (221.6)^a^ |  |
| VESTED | 50 weeks postpartum | DTG + FTC/TAF | 217 | 27 (22, 32) | 217 (100) | 467 (324, 624) | 2.9 (2.2, 3.8) |
|  |  | EFV + FTC/TDF | 211 | 27 (23, 32) | 211 (100) | 439 (300, 616) | 3.1 (2.3, 3.7) |
| REALITY | 48 | RAL + NNRTI/2NRTI | 902 | 35 (29, 42) | 423 (47) | 38 (16, 64) | 5.39 (4.97, 5.76) |
|  |  | NNRTI/2NRTI | 903 | 36 (29, 42) | 421 (47) | 36 (16, 61) | 5.40 (4.98, 5.80) |
| Reflate TB | 48 | RAL + 3TC/TDF | 51 | 37 (31, 44) | 16 (31) | 115 (50, 213) | 4.9 (4.4, 5.4) |
|  |  | EFV + 3TC/TDF | 51 | 35 (29, 45) | 12 (24) | 129 (45, 308) | 5.0 (4.5, 5.5) |
| Reflate TB 2 | 48 | RAL + 3TC/TDF | 230 | 34 (28, 42) | 92 (40) | 99 (39, 239) | 5.5 (5.0, 5.8) |
|  |  | EFV + 3TC/TDF | 227 | 37 (30, 43) | 90 (40) | 108 (35, 238) | 5.5 (5.0, 5.9) |
| ACTG A5257 | 96 | RAL + FTC/TDF | 603 | 36^c^ | 148 (25) | 304^d^ | 4.66^c^ |
|  |  | DRV/r + FTC/TDF | 601 | 37^c^ | 143 (24) | 310^d^ | 4.61^c^ |
| STARTMRK | 156 | RAL + FTC/TDF | 281 | 37 (19, 67)^a^ | 54 (19) | 212 (1, 620)^a^ | 5.1 (3.0, 6.0)^a^ |
|  |  | EFV + FTC/TDF | 282 | 36 (19, 71)^a^ | 51 (18) | 204 (4, 807)^a^ | 5.0 (4.0, 6.0)^a^ |
| NEAT001 | 96 | RAL + DRV/r | 401 | 37 (31, 45) | 49 (12) | 340 (260, 394) | 4.78 (4.30, 5.17) |
|  |  | DRV/r + FTC/TDF | 404 | 39 (31, 46) | 46 (11) | 325 (248, 401) | 4.75 (4.32, 5.12) |
| GS-US-236-0102 | 48 | EVG + COBI/FTC/TDF | 348 | 37 (29, 45) | 41 (12) | 376 (276, 487) | 4.75 (4.32, 5.15) |
|  |  | EFV + FTC/TDF | 352 | 38 (30, 45) | 36 (10) | 383 (268, 479) | 4.78 (4.37, 5.15) |
| 1. Median (range) was reported instead of median (IQR). 2. Mean (standard deviation) was reported instead of median (IQR). 3. Median was reported without range or IQR. 4. Mean was reported without standard deviation. | | | | | | | |
| *IQR, interquartile range; DTG, dolutegravir; ABC, abacavir; 3TC, lamivudine; EFV, efavirenz; TDF, tenofovir disoproxil; FTC, emtricitabine; TAF, tenofovir alafenamide; NRTI, nucleoside reverse transcriptase inhibitor; NNRTI, non-nucleoside reverse transcriptase inhibitor; RAL, raltegravir; EVG, elvitegravir; COBI, cobicistat; DRV/r, ritonavir boosted darunavir.* | | | | | | | |

**Table 3. Risk of bias assessment**

| **Risk of bias assessment for the primary outcome (IRIS)** | | | | | | |
| --- | --- | --- | --- | --- | --- | --- |
|  | **Bias** | | | | | |
| **Study** | **Randomisation process** | **Deviation from intended interventions** | **Missing outcome data** | **Measurement of the outcome** | **Selection of the reported results** | **Overall** |
| INSPIRING | Low | Low | Low | Low | Low | Low |
| DolPHIN-2 | Low | Low | Low | Low | Some concerns | Some concerns |
| NAMSAL | Low | Some concerns | Low | High | Some concerns | High |
| ADVANCE | Low | Low | Low | Some concerns | Some concerns | Some concerns |
| SINGLE | Low | Low | Low | High | Some concerns | High |
| VESTED | Low | Low | Low | High | Some concerns | High |
| Advanz-4 | Some concerns | Some concerns | High | High | Some concerns | High |
| Reflate TB | Low | Low | Low | Some concerns | Some concerns | Some concerns |
| Reflate TB 2 | Low | Low | Low | Some concerns | Low | Some concerns |
| REALITY | Low | Low | Low | Low | Low | Low |
| ACTG A5257 | Some concerns | Low | Low | High | Some concerns | High |
| STARTMRK | Low | Low | Low | Some concerns | Some concerns | Some concerns |
| NEAT001 | Low | Low | Low | High | Some concerns | High |
| GS-US-236-0102 | Low | Low | Low | Some concerns | Some concerns | Some concerns |
| **Risk of bias assessment for the secondary outcome (paradoxical TB-IRIS)** | | | | | | |
|  | **Bias** | | | | | |
| **Study** | **Randomisation process** | **Deviation from intended interventions** | **Missing outcome data** | **Measurement of the outcome** | **Selection of the reported results** | **Overall** |
| INSPIRING | Low | Low | Low | Low | Low | Low |
| Reflate TB | Low | Low | Low | Some concerns | Some concerns | Some concerns |
| Reflate TB 2 | Low | Low | Low | Some concerns | Low | Some concerns |
| *IRIS, immune reconstitution inflammatory syndrome; TB-IRIS, tuberculosis-associated immune reconstitution inflammatory syndrome.* | | | | | | |

**Table 4. Summary of findings**

| **Summary of findings. InSTI regimens compared to non-InSTI regimens for ART-naïve HIV-positive patients** | | | | | | |
| --- | --- | --- | --- | --- | --- | --- |
| **InSTI regimens compared to non-InSTI regimens for ART-naïve HIV-positive patients** | | | | | | |
| **Patient or population:** ART-naïve HIV-positive patients  **Setting:** any settings  **Intervention:** InSTI regimens  **Comparison:** non-InSTI regimens | | | | | | |
| Outcomes | **Anticipated absolute effects^*^** (95% CI) | | Relative effect (95% CI) | № of participants (studies) | Certainty of the evidence (GRADE) | Comments |
|  | **Risk with non-InSTI regimens** | **Risk with InSTI regimens** |  |  |  |  |
| IRIS | 37 per 1,000 | **35 per 1,000** (28 to 43) | **RR 0.93** (0.75 to 1.14) | 8696 (14 RCTs) | ⨁⨁⨁◯ Moderate^a^ | InSTI regimens probably result in little to no difference in IRIS risk. |
| Paradoxical TB-IRIS | 71 per 1,000 | **45 per 1,000** (24 to 84) | **RR 0.64** (0.34 to 1.19) | 674 (3 RCTs) | ⨁⨁◯◯ Low^b,c^ | InSTI regimens may reduce risk of paradoxical TB-IRIS. |
| ***The risk in the intervention group** (and its 95% confidence interval) is based on the assumed risk in the comparison group and the **relative effect** of the intervention (and its 95% CI).  **CI:** confidence interval; **RR:** risk ratio; **InSTI:** integrase strand transfer inhibitor; **ART:** antiretroviral therapy; **HIV:** human immunodeficiency virus; **IRIS:** immune reconstitution inflammatory syndrome; **TB-IRIS:** tuberculosis-associated immune reconstitution inflammatory syndrome; **RCT:** randomized controlled trial. | | | | | | |
| **GRADE Working Group grades of evidence** **High certainty:** we are very confident that the true effect lies close to that of the estimate of the effect. **Moderate certainty:** we are moderately confident in the effect estimate: the true effect is likely to be close to the estimate of the effect, but there is a possibility that it is substantially different. **Low certainty:** our confidence in the effect estimate is limited: the true effect may be substantially different from the estimate of the effect. **Very low certainty:** we have very little confidence in the effect estimate: the true effect is likely to be substantially different from the estimate of effect. | | | | | | |
| a. Downgraded one level for serious risk of bias due to overall high risk of bias in six studies.  b. Downgraded one level for serious risk of bias due to some concerns for the overall risk of bias in two studies.  c. Downgraded one level for serious imprecision due to few events. The 95% confidence interval is wide and includes no effect. | | | | | | |

**Table 5. IRIS events reported in included studies**

|  |  | **InSTI arm** | | | **Control arm** | | |  |
| --- | --- | --- | --- | --- | --- | --- | --- | --- |
| **Study** | **N** | **Regimen** | **IRIS events** | **N** | **Regimen** | **IRIS events** | **N** | **RR (95% CI)** |
| SINGLE | 833 | DTG + ABC/3TC | 1 (<1%) | 414 | EFV + TDF/FTC | 2 (<1%) | 419 | 0.51 (0.05, 5.56) |
| DolPHIN-2 | 268 | DTG + 2NRTI | 1 (<1%) | 137 | EFV + 2NRTI | 0 | 131 | 2.87 (0.12, 69.82) |
| Advanz-4 | 101 | DTG + ABC/3TC | 5 (9.6%) | 52 | DRV/r + ABC/3TC | 6 (12%) | 49 | 0.79 (0.26, 2.41) |
| INSPIRING | 113 | DTG + 2NRTI | 5 (7%) | 69 | EFV + 2NRTI | 4 (9%) | 44 | 0.80 (0.23, 2.81) |
| NAMSAL | 613 | DTG + 3TC/TDF | 0 | 310 | EFV + 3TC/TDF | 2 (<1%) | 303 | 0.20 (0.01, 4.06) |
| ADVANCE | 702 | DTG + TAF/FTC | 0 | 351 | EFV + TDF/FTC | 3 (1%) | 351 | 0.14 (0.01, 2.76) |
| VESTED | 428 | DTG + FTC/TAF | 0 | 217 | EFV + FTC/TDF | 1 (<1%) | 211 | 0.32 (0.01, 7.91) |
| REALITY | 1805 | RAL + NNRTI + 2NRTI | 89 (9.9%) | 902 | NNRTI + 2NRTI | 86 (9.5%) | 903 | 1.04 (0.78, 1.37) |
| Reflate TB | 102 | RAL + 3TC/TDF | 2 (4%) | 51 | EFV + 3TC/TDF | 5 (10%) | 51 | 0.40 (0.08, 1.97) |
| Reflate TB 2 | 459 | RAL + TDF/3TC | 25 (11%) | 229 | EFV + TDF/3TC | 38 (17%) | 230 | 0.66 (0.41, 1.06) |
| ACTG A5257 | 1204 | RAL + FTC/TDF | 1 (<1%) | 603 | DRV/r + FTC/TDF | 0 | 601 | 2.99 (0.12, 73.25) |
| STARTMRK | 563 | RAL + FTC/TDF | 19 (7%) | 281 | EFV + FTC/TDF | 13 (5%) | 282 | 1.47 (0.74, 2.91) |
| NEAT001 | 805 | RAL + DRV/r | 2 (<1%) | 401 | DRV/r + FTC/TDF | 1 (<1%) | 404 | 2.01 (0.18, 22.13) |
| GS-US-236-0102 | 700 | EVG + COBI/FTC/TDF | 0 | 348 | EFV + FTC/TDF | 1 (<1%) | 352 | 0.34 (0.01, 8.25) |
| *IRIS, immune reconstitution inflammatory syndrome; InSTI, integrase strand transfer inhibitor; RR, relative risk; DTG, dolutegravir; ABC, abacavir; 3TC, lamivudine; EFV, efavirenz; TDF, tenofovir disoproxil; FTC, emtricitabine; TAF, tenofovir alafenamide; NRTI, nucleoside reverse transcriptase inhibitor; NNRTI, non-nucleoside reverse transcriptase inhibitor; RAL, raltegravir; EVG, elvitegravir; COBI, cobicistat; DRV/r, ritonavir boosted darunavir.* | | | | | | | | |
